# Exploiting the power of LINE-1 retrotransposon mutagenesis for identification of genes involved in embryonic stem cell differentiation

**DOI:** 10.1007/s12015-014-9500-9

**Published:** 2014-03-11

**Authors:** Nibedita Lenka, Shruthi Krishnan, Philip Board, Danny Rangasamy

**Affiliations:** 1 National Centre for Cell Science, Pune, 411007 India; 2 John Curtin School of Medical Research, The Australian National University, Canberra, Australian Capital Territory 2601 Australia

**Keywords:** Retrotransposon, Transgenesis, Embryonic stem cells, Loss-of-function, Gene disruption, Differentiation, Essential genes

## Abstract

Identifying the genes or epigenetic factors that control the self-renewal and differentiation of stem cells is critical to understanding the molecular basis of cell commitment. Although a number of insertional mutagenesis vectors have been developed for identifying gene functions in animal models, the L1 retrotransposition system offers additional advantages as a tool to disrupt genes in embryonic stem cells in order to identify their functions and the phenotypes associated with them. Recent advances in producing synthetic versions of L1 retrotransposon vector system and the optimization of techniques to accurately identify retrotransposon integration sites have increased their utility for gene discovery applications. We have developed a novel episomal, nonviral L1 retrotransposon vector using scaffold/matrix attachment regions that provides stable, sustained levels of retrotransposition in cell cultures without being affected by epigenetic silencing or from some of the common problems of vector integration. This modified vector contains a GFP marker whose expression occurs only after successful gene disruption events and thus the cells with disrupted genes can be easily picked for functional analysis. Here we present a method to disrupt gene function in embryonic stem cells that aid in the identification of genes involved in stem cell differentiation processes. The methods presented here can be easily adapted to the study of other types of cancer stem cells or induced pluripotent stem cells using the L1 retrotransposon as an insertional mutagen.

## Introduction

Embryonic stem cells (ESCs) offer great hope for the treatment of genetic and malignant diseases and their ability to differentiate into any specific cell type of the body is an area of intense research. In vitro, ESCs can be maintained in an undifferentiated state for prolonged periods of time, while retaining their competence to differentiate into various cell types [1]. Additionally, by manipulating their growth conditions, ESCs can be induced to differentiate into specific cell types of interest. In recent years, much progress has been made in optimizing ESC differentiation protocols using cocktails of growth factors [2], chemical compounds [3], or by using tissue-specific promoters to enrich selected populations of cells [4]. ESCs can be maintained as undifferentiated cells in the presence of the cytokine leukemia inhibitory factor (LIF), which directs ESC self-renewal through the activation of the transcriptional factor, STAT3 [5]. Upon removal of LIF, ESCs are induced to differentiate into spheroid cell aggregates termed embryoid bodies, recapitulating early embryonic developmental processes. Another extrinsic factor involved in directing ESC self-renewal is the bone morphogenic protein BMP4, which induces the expression of inhibitors of differentiation genes via Smad-signaling [6]. In addition to LIF and BMP4, a number of intrinsic factors including Oct4, Nanog, Sox2, mitogen-activating protein kinase (MAPK) and members of the Wnt signaling pathway form a complex regulatory network regulating the determination of ESC cell fate [7, 8]. Recent discoveries of new pluripotency factors, epigenetic modifications and miRNAs suggest that multiple genetic and epigenetic modulators influence ESC differentiation [9, 10]. However, our current understanding of the genes or epigenetic factors that control the self-renewal and differentiation of ESCs is far from complete, and thus identifying these factors is critical for understanding the molecular basis of cell commitment and that might also suggest novel strategies for the directed differentiation of ESCs.

Loss-of-function studies are a powerful method for identifying genes by disrupting their functions and investigating the downstream functional consequences of these manipulations. In gene discovery approaches, many agents, including chemical mutagens, viruses, and small interfering RNAs are used to disrupt genes to identify their functions and the phenotypes associated with them. The most commonly used chemical mutagen is the alkylating agent, *N*-ethyl-*N*-nitrosourea (ENU), which can produce point mutations in male spermatogonial stem cells resulting in loss- or gain-of-gene function [11]. However, screens for mutations of interest in such systems require the breeding of at least one generation of mice. A dominant mutation that results in visible phenotypes such as changes in coat color, morphology, or behavior can be easily identified in the first generation of mice breeding. On the other hand, recessive mutations require multiple generations of mice breeding in order to understand the functions of affected gene and its responses to developmental pathways, if any [12, 13]. In both cases, a mutation generated by ENU is identified by DNA sequencing. Furthermore, the applications of ENU approaches are strictly limited to the production of germline transgenic animals.

To overcome these limitations, a number of insertional mutagenic systems have been developed for generating loss-of-function mutations [14]. Early methods relied on the use of retroviral vectors derived from murine leukemia viruses, which integrate into dividing cells and disrupt expression of nearby genes. However, this approach is limited by a number of shortcomings including a non-uniform distribution of retroviral integration and the necessity of generating adult mice for functional analysis [15, 16]. In recent years, a number of DNA transposons such as the *Sleeping Beauty* (SB) and *PiggyBac* (PB) vectors have been developed for identification of genes involved in cancer cells and mice models [17–19]. DNA transposons require a corresponding protein component, called a transposase, for their transpositional activity. Recently, the *SB* transposon has been extensively modified by the conditional activation of the transposase for tissue-specific transposition activity [20]. Despite certain improvements and increased transgenic efficiency, these systems still require co-delivery of the transposon with the transposase-encoding DNA for gene integration to occur [21]. Low transfer efficiency and a lack of sustained transposase expression have also been reported to occur in some cases of cell culture [22, 23]. Although the *SB* and *PB* systems are commonly used, other retrotransposons such as the long interspersed element (LINE-1 or L1 retrotransposon) are currently being exploited as an alternative tool for loss-of-gene function screen.

The L1 vector is a single-component retrotransposition system that offers significant advantages over other DNA transposon vectors due to being less demanding of laboratory and technical conditions. Unlike the *SB* and *PB* transposons, which work by a “cut-and-paste” mechanism, L1 mobilizes itself to new genomic locations by a “copy and paste” mechanism thus offering an unlimited source of insertional mutagens for gene knockout throughout the genome [24, 25]. In addition, L1 insertion is stable and permanent in all the progeny of the cells in which it has integrated and thus the inserted sequence itself serves as a ‘molecular tag’ to identify the disrupted genes within a target genome. The requirements for L1 retrotransposition are the presence of two L1-encoded proteins (ORF1p and ORF2p). ORF1p encodes a protein with RNA-binding activity, while ORF2p encodes an endonuclease activity with a reverse transcriptase for mobilization. The endonuclease generates a random nick in the target DNA, and the reverse transcriptase uses the nicked DNA to prime reverse transcription of the L1 RNA, followed by stable integration of the L1 DNA copy into the new genomic location. We have used the cytomegalovirus (CMV) immediate early promoter to drive the expression of L1-encoded proteins, which have been reported to be transcriptionally active in a variety of ES cells and other cells [26]. For easy detection of gene disruption, the L1 vector contains an engineered GFP marker at the 3′-UTR end of the poly-A signal [27, 28]. This GFP gene is disrupted by 960 bp from a γ–globin intron in an antisense orientation and is transcribed as a single fusion transcript due to the presence of the splicing sites in the intron sequences. This arrangement ensures that GFP expression occurs only after the successful integration of the L1 copy into a new location thereby helping to detect a gene disruption event in living ESCs without cell staining. Thus the cells harboring a new L1 integration can be easily identified using fluorescence microscopy and inverse PCR-based techniques.

In an effort to improve the L1 system, several groups have developed a *codon-optimized* L1 retrotransposon, which has been successfully used in mouse models to identify genes that are involved in neurogenesis and brain development [25]. In addition, a recent development of a conditionally regulated L1 expression has increased their utility for generating tissue-specific loss-of-function mutations in vivo [29]. Recently, we used the human L1 element (L1_RP_) that contains a GFP marker to generate insertional mutations in mouse ESCs [30]. To ensure the stability and the integrity of the L1 vector, we added a DNA fragment containing scaffold/matrix attachment regions (S/MARs) in the vector backbone. The inclusion of S/MARs has three significant advantages. First, it stably maintains a single copy of the L1 vector in ES cells, which is not subject to silencing, or loss of the vector, even in the absence of antibiotic G418 selection [31]. A second advantage is the low levels of retrotransposition activity. This is particularly important in stem cells since high levels of retrotransposition or a short burst of L1 overexpression often results in too many L1 disruptions within a single cell, leading to difficulties in screening the colonies. Third, the vector contains a neomycin/G418 resistance gene that can be used to select transfected ESC clones. These modifications of L1 system make it an ideal tool in ESC gene discovery applications.

Implementing the L1-based loss-of-function screening approach requires three key steps in order to identify the genes involved in ESC differentiation and self-renewal. The first step is the delivery of the L1 vector into ESCs for insertional mutagenesis. In recent years, much progress has been made in optimizing ESC transfection protocols using electroporation, liposome, or nucleofection to achieve high transfection efficiencies [32]. Several commercial kits are also available for this step. We have found that one can achieve 30–35 % transfection efficiency with the use of Amaxa nucleofector reagents without affecting the properties of mouse ESCs or inducing cytotoxicity. Second is screening for loss-of-function phenotypes using the dependency of ESCs on LIF. Self-renewal of ESCs is traditionally maintained by culturing them in the presence of LIF. Upon removal of LIF, ESCs commit to differentiation under the influence of serum factors. Disruptions in genes that regulate ESC commitment or repression of pluripotency are anticipated to reduce dependency on LIF. This strategy allows us to identify the endogenous genes that potentially regulate ESC renewal. Undifferentiated ESCs normally maintain an open chromatin structure with reduced global levels of DNA methylation compared to differentiated ESCs or lineage-committed cells [33]. Under these conditions, expression of the L1 retrotransposon is expected to occur resulting in insertion of L1 copy into the host genome. When L1 inserts into a gene, the protein encoded by that gene is likely to be effectively truncated and its function disrupted. If the disrupted gene is essential for cell differentiation, then ESCs may remain in an undifferentiated state even after being subjected to differentiation-inducing conditions, such as the withdrawal of LIF. To facilitate detection of gene disruption events, the L1 vector contains a GFP reporter that is expressed only when the newly synthesized L1 DNA copy is successfully integrated into a new genomic location. Using fluorescence microscopy, each GFP-positive clone can be individually picked for further morphological analysis. Third is the identification of disrupted genes using the inverse PCR method and confirming that a defective ESC clone is a result of gene disruption. The inverse PCR assay is a simple method for identifying the disrupted gene that flanks an L1 integration site. Additional information about the methodology can be found in elsewhere [25, 30].

By using the modified L1 vector system, we recently screened about 50 ESC clones in a single 100-mm petriplate [30]. Since the expression level of this vector system is relative low in comparison to *codon-optimized* L1 vectors, 41 ESC clones did not show GFP expression. In total, we isolated 9 individual GFP-positive clones of which 4 of them contained the new insertions in the noncoding intergenic regions as measured by inverse PCR method. These clones also became differentiated within 5 days, similar to the wild-type ESCs, when cultured in media without LIF. While one clone contained the multiple L1 insertions, the remaining 4 ESC clones displayed a gene-specific mutation, including the published Arp6-actin related gene (*Actr6*). The integration of a new insertion of L1 copy into a gene truncates the protein encoded by that gene, resulting in loss-of-function mutation. Notably, all these mutated ESCs remained undifferentiated state, irrespective of the presence or absence of LIF and MEFs. To further verify the function of each gene in ESC differentiation, we used an RNAi approach to knockdown the gene function in wild-type ESCs, followed by confirmation of defectiveness in the differentiation processes [30]. Strikingly, one of the identified genes is Toll-interacting protein (*Tollip*) whose function has never been reported in cell differentiation. The integration of L1 DNA copy at the exon 2 of the *Tollip* gene made the ESCs inability to synthesize the functionally active gene product.

**Fig. 1:**
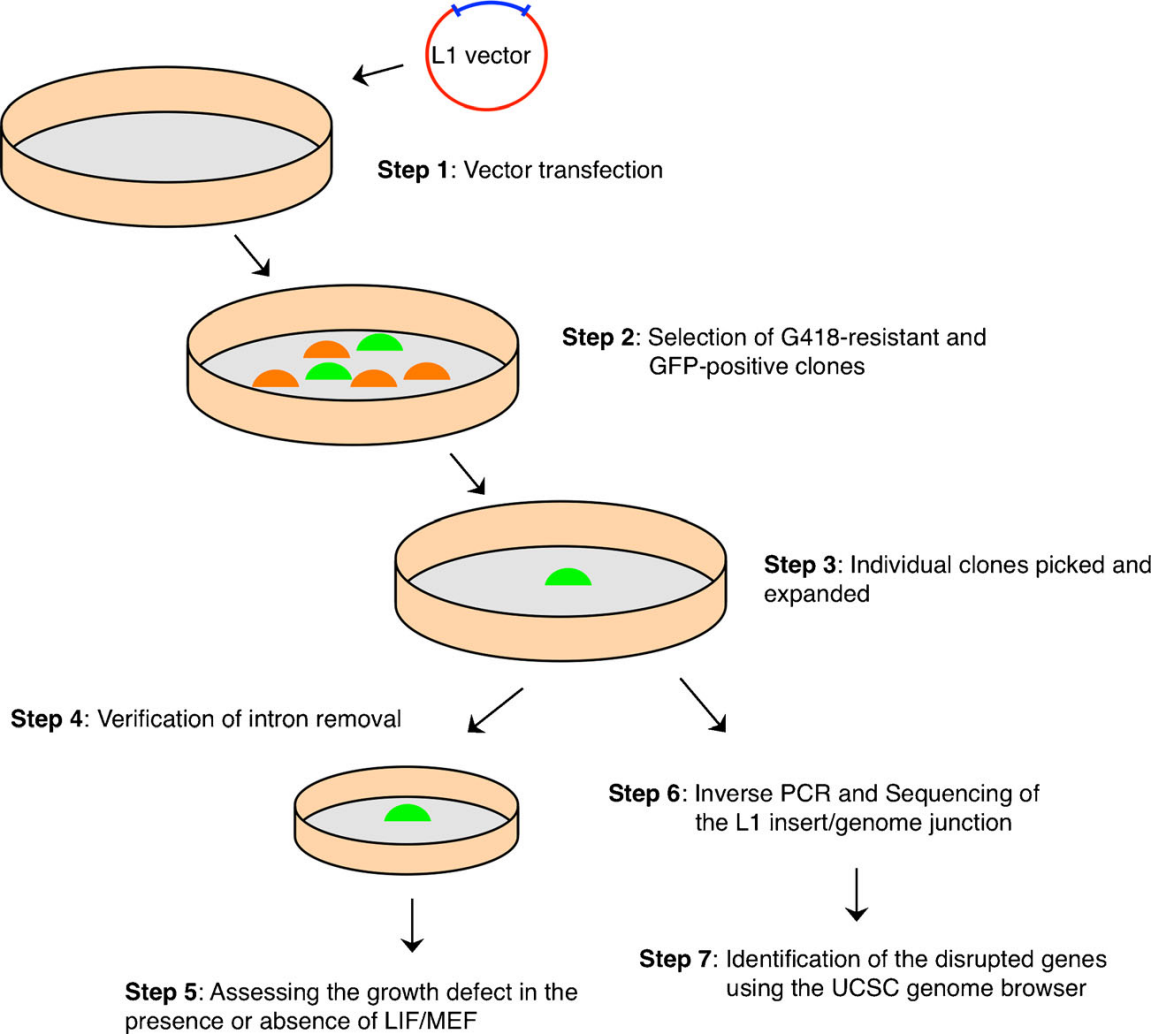
Outline of the steps involved in L1-mediated loss-of-function screens to find genes associated with stem cell differentiation and self-renewal

Here we present a simple and reproducible method for using the L1 retrotransposon system to generate a loss-of-function genetic screen in a mouse ESC model system. This method involves an initial transfection of vector and isolates ESCs in the classical medium with the use of LIF (Fig. 1). We show that an L1 vector, coupled with GFP reporter expression, can facilitate the identification of genes that are implicated in ESC differentiation using inverse PCR analysis. In addition, it has the added advantage of allowing simultaneous monitoring of loss-of-gene function and its associated cellular states and differentiation processes with minimal genetic manipulation. The methods presented here can be easily adapted to the study of other types of stem cells, induced pluripotent stem (iPS) cells, or cancer stem cells to identify the genes that are responsive to certain cell growth conditions or morphology.

## Materials

1. Mouse ES cell line D3 (SV129 origin; ATCC) or ESCs derived from C57BL/6.
2. Mouse embryonic fibroblasts (MEF): derived from E12.5–14.5 day old mouse embryos - either wild type or Neo^R^ strain (Jackson Labs).
3. Plastic ware: Tissue culture dishes (35 and 100 mm; 24-, 48-, 96-well flat bottom) and Cryovials (1.5 ml).
4. ESC maintenance medium: Dulbecco’s Modified Eagle Medium (DMEM) (Invitrogen) supplemented with 2 mM L-Glutamine, 100U/ml Penicillin, 100 μg/ml streptomycin, 1× Non-essential Amino Acid (all from Invitrogen), 0.1 mM β-Mercaptoethanol (Sigma) and 15 % FBS (ES cell-qualified, Hyclone). Store at 4 °C. Add 1000U/ml leukemia inhibitory factor (LIF) directly to the dish after seeding the cells.
5. Fibroblast medium: DMEM supplemented with 10 % FBS, 2 mM L-Glutamine, 100U/ml Penicillin, and 100 μg/ml streptomycin. Store at 4 °C.
6. 0.25 % Trypsin-1 mM EDTA (Invitrogen).
7. Geneticin (G418) (Sigma): The stable clones are selected by exposing ESCs to 300–400 μg/ml G418 on day 2 post-transfection. The G418 resistant clones are picked up after 7 days of antibiotic exposure.
8. Phosphate buffered saline (PBS)
9. Gelatin (Sigma)
10. Nucleofector kit for ES cells (Amaxa)
11. Topo-XL vector or any type of vector for PCR cloning
12. Biosafety cabinet
13. Inverted fluorescent microscope
14. Water jacketed CO_2_ incubator
15. Hemocytometer/Neubauer chamber
16. Centrifuge and centrifuge tubes
17. Nucleofector device (Amaxa Biosystems)
18. Thermal cycler or PCR machine

## Methods

### ESCs maintenance and passaging

ESCs are normally maintained in an undifferentiated state by culturing them on a monolayer of mitotically inactive fibroblast feeders [derived either by Mitomycin C treatment [34] or by exposure to γ-irradiation]. An alternative method is to seed the cells on a 0.1 % gelatin-coated dish and maintain them in the presence of 1000U/ml LIF in ESC maintenance medium. However, prior to differentiation we prefer to maintain the ESCs without the MEF feeders on gelatin-coated dishes for a minimum of 2–3 passages. The entire procedure is carried out aseptically in a biosafety cabinet.

1. Remove the medium from the dish containing ~80 % confluent ESCs and wash with 1× PBS.
2. Dislodge the cells from the culture dish with 0.25 % Trypsin-EDTA by incubating for about 1 min and rock gently to obtain single cells. To inactivate trypsin, add twice the volume of the maintenance medium.
3. Collect the cells in a fresh tube and pellet down by centrifugation at 1000 rpm for 5 min. Aspirate the supernatant, resuspend the cell pellet in the maintenance medium and take the cell count using the hemocytometer.
4. Seed ~1 × 10^5^ ESCs in a 35 mm dish with or without feeders (the seeding density will depend on the diameter of the dish and also the doubling time of cells such that ESCs should attain at least 70–80 % confluence within a 48 h time period) using maintenance medium supplemented with 1000U/ml LIF and incubate the dish in a humidified incubator at 37 °C and 5 % CO_2_ for propagation and maintenance.

### Transfection of ESCs

5. The day before transfection, precipitate 5–10 μg of L1 retrotransposon vector with absolute ethanol and incubate overnight at −20 °C (Note 1).
6. Grow ESCs until they are 70–80 % confluent and process them to obtain a single cell suspension following steps 1–3 for nucleofection.
7. Prepare the nuclefection mix by adding nuclefection solution and the supplement from the nucleofection kit designated for ESCs at a ratio of 4.5:1.
8. Take 0.2–2 × 10^6^ cells and wash with PBS and suspend the cell pellet in 90 μl of nucleofection mix.
9. Meanwhile, spin down the DNA from step 5 at 13000× *g* for 15 min and wash the DNA pellet with 70 % ethanol. Carefully aspirate 70 % ethanol and leave the tube open in the biosafety cabinet for 2–3 min to dry the DNA pellet (over-drying of DNA pellet should be avoided) (Note 2).
10. Dissolve the vector DNA in 10 μl of nucleofection mix, add to cells from step 8, mix well and transfer the entire contents to the nucleofector cuvette without introducing air bubbles.
11. Place the cuvette in the nucleofector device and carry out nucleofection using programme A-23.
12. Immediately transfer the contents to a 100 mm dish (either gelatin-coated or with a monolayer of mitotically inactive Neo^R^ MEF) containing pre-warmed ESC maintenance medium supplemented with 1000U/ml LIF. Incubate the cells at 37 °C inside a humidified 5 % CO_2_ incubator.

### Selection of stable ESC clones and colony picking

13. Initiate G418 selection of ESCs (300 μg/ml, optimization may be required depending on the mouse ES cell line used), 48 h post-transfection and change medium every day during the initial selection period (3–4 days) and subsequently every alternate day to remove the dead cells from the culture dish.
14. Monitor for GFP expression in the resistant clones under the inverted fluorescent microscope and mark the colonies to be picked up after 7 days of G418 selection (the colonies at this stage should look morphologically distinct and visible to the naked eye) (Note 3).
15. Place an inverted microscope in the biosafety cabinet. Aspirate the medium from the dish, add 1× PBS and place it on the microscope stage.
16. Manually pick the marked clones individually and transfer to a 96 multiwell dish (one colony/well) already containing 50 μl of Trypsin-EDTA.
17. Trypsinize the cells gently for single cell preparation and subsequently inactivate trypsin by adding 100 μl of ESC maintenance medium. Transfer the cells individually from each well to a 48 multiwell dish (either gelatin-coated or containing a monolayer of Neo^R^ MEF feeders) and maintain the clones in the presence of 300 μg/ml G418 throughout.
18. Incubate the clones at 37 °C in a humidified incubator with 5 % CO_2._ Propagate the confluent clones individually in a 24-well dish till they attain confluency. Replenish the maintenance medium supplemented with LIF and G418 every 2 days. Examine each well and mark the confluent wells for splitting/freezing.

### Verification of gene disruption

**Fig. 2:**
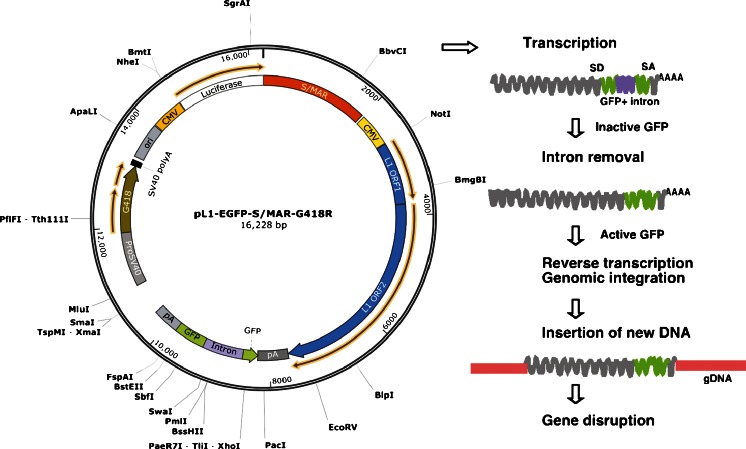
Schematic diagrams of the pL1-EGFP-S/MAR retrotransposon vector system. GFP is co-expressed as a single fusion transcript due to the presence of the splicing sites in the intron sequences. This arrangement ensures that GFP expression occurs only after successful insertion of an L1 copy into a gene. The vector map (*left panel*) and the flowchart of L1 retrotransposition event or gene disruption (*right panel*) are shown. *SD* splice donor; *SA* splice acceptor; *CMV* cytomegalovirus early promoter; *S/MAR* scaffold/matrix attachment regions; *GFP* GFP reporter gene

**Fig. 3:**
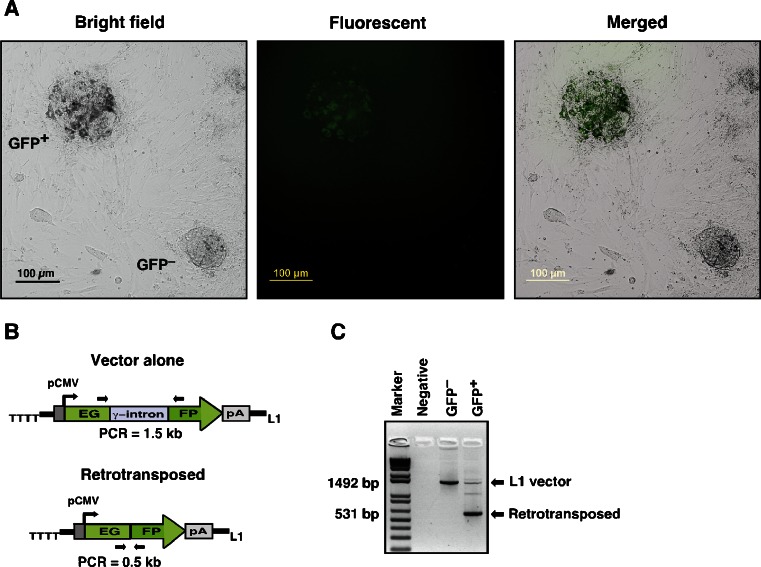
Generation of loss-of-function screens using the L1 retrotransposon system. Mouse ESCs are transfected with L1 vector and allowed to proliferate for 7 days in the presence of LIF to initiate gene disruption. **a** A representative result of ESC colonies expressing GFP and no GFP expression formed by transfected cells. **b** Rationale for the PCR analysis. The 1.5 kb PCR product represents the original L1 vector harboring the intron-containing GFP, while 0.5 kb PCR product indicates a gene disruption and the loss of the intron. **c** A representative result of ESCs analyzed by PCR amplification. The symbol – and + represents the GFP-positive and GFP-negative ESC colonies. Negative, untransfected (control) ESCs; Marker, 1 kb-plus DNA marker

GFP is co-transcribed as a single fusion transcript due to the presence of the splicing sites in the introns of the L1 vector. This arrangement ensures that GFP expression occurs only after L1 expression, intron splicing, reverse transcription and integration of the L1 copy into the genomic DNA (Fig. 2). Therefore, no GFP is expressed unless the newly synthesized L1 is integrated into a genomic location. Thus, detection of GFP expression in clones suggests successful gene disruption has taken place in ESCs. Additionally a simple PCR amplification of the intron-GFP junctions gives a quick readout of whether a given cell has a new insert in their genome. See Fig. 3a for a typical example of disrupted gene in mouse ESC screening.

19. Isolate genomic DNA from the selected ESC clones using a QIAamp DNA kit (Qiagen).
20. Perform PCR amplification with forward (5′-TATTGCCGATCCCCTCAGAAGA-3′) and reverse (5′-CAAGGACGACGGCAACTACAAG-3′) primers that correspond to the GFP sequence flanking the γ-globin intronic sequences (Fig. 3b).
21. Visualize the amplified product on an agarose gel. While the 1.49 kb PCR product represents the original L1 vector containing the intron-containing GFP (i.e., no retrotransposition), the intron splicing subsequent to L1 insertion would give a 531 bp PCR product reflecting the selection of positive clones (Fig. 3c) (Note 4).

### Identification of disrupted genes

Inverse PCR is commonly used to amplify the flanking sequences of the disrupted genes to enable precise determination of the gene’s identity using the UCSC Genome Browser. In this assay, the forward primer binds at just upstream of the polyA signal of the GFP gene and the reverse primer at downstream of the GFP gene, which allows for specific amplification of the flanking sequences of the L1 integration sites.

22. Digest 1 μg of genomic DNA with Ssp1 restriction enzyme at 37 °C and then purify DNA using a QIAquick PCR purification kit (Qiagen).
23. Carry out self-ligation of the digested DNA using 20 units of T4 DNA ligase in a total volume of 100 μl.
24. Use 20 μl (i.e., 200 ng) of the ligation mixture directly as the DNA template for the PCR reaction with forward (5′-TGATAAGATACATTGATGAGTTTGGA-3) and reverse (5′-TATATCTCCCAATGCTATCC-3′) primers in a total volume of 50 μl. Cycling conditions are 1× (94 °C, 2 min), 10× (94 °C, 30 s; 58 °C, 30 s; 72 °C, 1 min), 25× ((94 °C, 20 s; 65 °C, 30 s; 72 °C, 1 min) followed by 1× (72 °C, 7 min).
25. Clone the amplified PCR product into a Topo-XL (Invitrogen) or pGEM (Promega) vector followed by DNA sequencing using M13 forward and reverse primers.
26. Identify the flanking sequences of the disrupted regions by BLAST search of the UCSC Mouse Genome Browser.

## Notes

1. It is important to use supercoiled plasmid DNA to increase transfection efficiency of ESCs, which can be easily checked by running a portion of plasmid DNA on a 1 % agarose gel. Supercoiled DNA migrates faster on a gel due to its conformation compared to the nicked or linear forms of plasmid. Most of the commercially available kits such as Qiagen or Sigma kit generally yield highly purified, supercoiled DNA, which works well for ESCs transfection.
2. The volume of nucleofection mix added to plasmid DNA should not exceed 10 % of the total reaction volume (10 μl for 100 μl reactions), as an excess of nucleofection solution might influence transfection results.
3. The transfected ESCs can be kept in an undifferentiated state for a period of 7 to 10 days in the presence of LIF and G418 to initiate insertional mutagenesis. When an L1 element is inserted into a new genomic location, there can be visible differences in the appearance of ESCs under fluorescence microscopy. The retrotransposed cells normally appear GFP-positive within 3–5 days of G418 selection. However, we have found that not all ESCs express a high GFP signal. In a pilot study we found that about 8–12 % of an ESC population display resistance to G418. Of which, except few, many of the retrotransposed cells express a faint GFP signal. This is mainly due to the presence of a single copy of the L1 vector and the low levels of retrotransposition activity. If GFP signal is too weak to differentiate it from that of the background under the fluorescence microscope, it is then required to perform PCR amplification of the GFP expression to confirm L1 insertions in the L1 stable cells.
4. Variability in GFP expression is the common problem encountered in L1-mediated loss-of-function screens, which is usually attributed to a newly integrated L1 copy in the host genomic DNA being subjected to the influence of chromosomal regulatory elements adjacent to the integration site [35]. Therefore PCR amplification of the GFP is required to identify and confirm that a gene has been disrupted by L1 insertion. By comparing the presence or absence of spliced GFP in these cells, one can confirm whether a gene disruption is indeed a result of L1 retrotranspositional activity. Figure 3 shows an example of an L1-disrupted gene from GFP-positive but not in GFP-negative ESCs. The second key step for successful implementation of the L1 system is in expanding the selected ESC clones and reassessing the clones morphology. If the genes are essential for differentiation, the disruption of those genes should give similar undifferentiated ESC morphology and should impair differentiation. To assess the resistance of ESCs to differentiation, cells from selected ESCs are seeded in gelatin-coated dishes using maintenance medium containing G418 and grown in parallel either in the presence or absence of LIF with monitoring of the cells under the inverted microscope for morphological changes. Clones showing differentiated morphology even in the presence of LIF often contain the disrupted genes that are important for ESC maintenance. Similarly clones showing undifferentiated morphology in the absence of LIF suggest that the disrupted genes may have a role during differentiation. Thus, to confirm the ability of selected clones to undergo differentiation, one can validate the undifferentiated or differentiated states of the ESCs by qPCR analysis or immunostaining the cells with undifferentiated markers such as alkaline phosphatase, Oct4, Nanog and SSEA-1. By comparing the presence or absence of markers in ESCs having a disrupted gene to wild-type ESCs, one can verify if the clones are resistant to cell differentiation.

## Abbreviations

ESCs: Embryonic stem cells
L1 or LINE-1: Long interspersed element-1
GFP: Green fluorescent protein
S/MARs: Scaffold/matrix attachment regions
FBS: Fetal bovine serum
LIF: Leukemia inhibitory factor
IPCR: Inverse polymerase chain reaction
